# CANCER, COGNITION, AND COMMUNICATION: A 2-DAY INTERPROFESSIONAL GERIATRIC PSYCHO-ONCOLOGY SYMPOSIUM

**DOI:** 10.1093/geroni/igz038.2382

**Published:** 2019-11-08

**Authors:** Natalie Gangai, Ruth Manna, Smita Banerjee, Rosario Costas Muniz, Christian Nelson, Beatriz Korc-Grodzicki

**Affiliations:** 1 Memorial Sloan Kettering Cancer Center, New York, United States; 2 Memorial Sloan Kettering Cancer Center, New York, New York, United States

## Abstract

Background: Most cancer deaths are in patients older than 65 years. Healthcare professionals (HCPs) caring for older adults with cancer must be equipped with skills to manage cognitive related changes and the nuances of communication with patients and caregivers. Methods: A two-day interprofessional symposium was developed to increase knowledge regarding 1) chemotherapy-related cognitive changes; 2) distress, delirium, dementia and depression in older cancer patients; 3) communication with patients with cognitive deficits and their caregivers; 4) decision making capacity. Presenters include geriatric medicine, geriatric psychiatry, occupational therapy and legal, ethics and communication experts. Day one centered on didactics with a complex case interprofessional discussion. Day two comprised of a communication skills training consisting of three modules: Geriatrics 101, Communication and Cognitive Deficits and Shared Decision Making. Participants role-played with simulated older adult patients and caregivers. Knowledge, self-efficacy and satisfaction were assessed. Results: A total of 75 people attended day one and 33 people attended day two. Most attendees were white (74.4%) and female (85.4%). Nurses (36.6%), social workers (29.3%), physicians (14.6%), others (19.5%) were represented. Mean knowledge increased (t=-3.23, df (13), p<0.05) from pre (M=0.83) to post (M=0.96). Mean self-efficacy in communication skills increased significantly across the three modules from 3.33 to 4.51 on a 5-point Likert scale (t=-6.40, df=23, p<0.001). Discussion: This two-day symposium shows an increase of knowledge and self-efficacy among HCPs caring for older adults. Skills related to cognitive changes and communication are essential to providing patient-centered care and making shared decisions with older adults and their caregivers.

